# ﻿The description of the first rock-dwelling species of butterfly lizard *Leiolepis* Cuvier, 1829 (Squamata, Agamidae) from the Khorat Plateau in northeastern Thailand

**DOI:** 10.3897/zookeys.1210.127557

**Published:** 2024-08-27

**Authors:** Pratyaporn Wanchai, Attapol Rujirawan, Matthew L. Murdoch, Akrachai Aksornneam, Pattarapon Promnun, Amanda Kaatz, Jeren J. Gregory, Eddie Nguyen, William Van Iderstein, Evan S. H. Quah, L. Lee Grismer, Jesse L. Grismer, Anchalee Aowphol

**Affiliations:** 1 Department of Biological Science, Faculty of Science, Ubon Ratchathani University, Warin Chamrap District, Ubon Ratchathani 34190, Thailand; 2 Animal Systematics and Ecology Speciality Research Unit, Department of Zoology, Faculty of Science, Kasetsart University, Bangkok 10900, Thailand; 3 Biodiversity Center, Kasetsart University, Bangkok 10900, Thailand; 4 Department of Biology, La Sierra University, 4500 Riverwalk Parkway, Riverside, California 92505, USA; 5 Geneus Genetics Co., Ltd., 37/1 Sukhumvit 101/1, Bang Chak, Phra Kanong District, Bangkok 10260, Thailand; 6 Institute for Tropical Biology and Conservation, Universiti Malaysia Sabah, Jalan UMS, 88400, Kota Kinabalu, Sabah, Malaysia; 7 Lee Kong Chian Natural History Museum, National University of Singapore, 2 Conservatory Drive, Singapore 117377, Singapore; 8 School of Biological Sciences, Universiti Sains Malaysia, 11800 Minden, Penang, Malaysia; 9 Department of Herpetology, San Diego Natural History Museum, PO Box 121390, San Diego, California, 92112, USA

**Keywords:** Conservation, Indochina, *Leiolepis*, phylogenetics, rock-dwelling

## Abstract

A new species of rock-dwelling *Leiolepis* is described from the Khorat Plateau in northeastern Thailand. *Leiolepisglaurung***sp. nov.** can be differentiated from all other sexual species of *Leiolepis* by a combination of having a black gular region with a wide medial yellow stripe, a yellow ventrum with black mottling, bright red to orange subcaudal coloration, having reduced to no expandable flanks, and having only one black transverse bar on the flanks. This is the first rocky habitat-adapted *Leiolepis*. *Leiolepisglaurung***sp. nov.** demonstrates numerous ecological adaptations to survive in these rocky habitats. *Leiolepis* are known for their expandable flanks with bright display colors, however *Leiolepisglaurung***sp. nov.** has reduced or no ability to expand its flanks. We hypothesize this is an adaptation to reduce their body diameter to better fit into smaller rocky burrows unlike the larger and deeper burrows constructed in looser soils by other *Leiolepis* species. This discovery increases the number of *Leiolepis* species in Thailand to six, and worldwide to 11.

## ﻿Introduction

**Figure 1.**
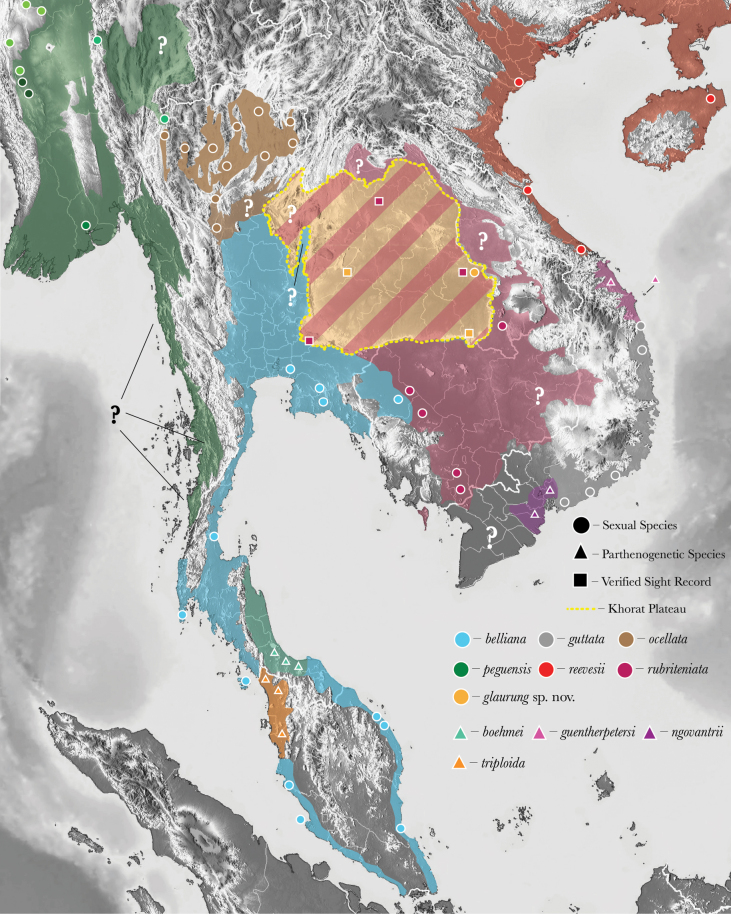
The distribution of all *Leiolepis* species and the localities of specimens used in this study. Question marks indicate an area where new populations could possibly occur. The different shades of the green circles for *Leiolepispeguensis* correspond to their phylogenetic positions in Fig. 2.

Butterfly lizards of the genus *Leiolepis* Cuvier, 1829 contain six sexual species, *L.belliana* (Hardwicke & Gray, 1827), *L.guttata* Cuvier, 1829, *L.ocellata* Peters, 1971, *L.peguensis* Peters, 1971, *L.reevesii* (Gray, 1831), and *L.rubritaeniata* Mertens, 1961, and four all-female parthenogenetic species, *L.boehmei* Darevsky & Kupriyanova, 1993, *L.guentherpetersi* Darevsky & Kupriyanova, 1993, *L.ngovantrii* Grismer & Grismer, 2010, and *L.triploida* Peters, 1971. These ten species represent a unique lineage of acrodont lizards that collectively range from southern China to Vietnam, Laos, Cambodia, Myanmar, Thailand, and south throughout the Malay Peninsula to Banda Island, Indonesia (Fig. 1). All species of *Leiolepis* are moderately sized (maximum snout–vent length 180 mm), diurnal omnivores that prefer coastal and inland savannah habitats with loose soil used to construct deep interconnected, subterranean burrow systems used for refugia and eggs deposition (Taylor 1963; Peters 1971; Cox et al. 1998; Aranyavalai et al. 2004; Grismer and Grismer 2010; Grismer 2011; Hartmann et al. 2012, 2013; Grismer et al. 2014). The six sexual species are sexually dimorphic and except for *L.guttata*, males have elongated ribs used to expand their flanks to display bright orange coloration (Hartmann et al. 2012). These expandable flanks and their colorations are used for courting females, warding off rival males, and anti-predator displays (Aranyavalai et al. 2004; Hartmann et al. 2012; Grismer et al. 2014). Conversely, the four parthenogenetic species are all similar in morphology and color pattern, resembling the coloration of their maternal ancestor, *L.guttata* (Schmitz et al. 2001; Grismer and Grismer 2010). Currently, four of the six sexual species, *L.belliana*, *L.ocellata*, *L.peguensis*, and *L.rubritaeniata* have populations within, or distributions that span much of Thailand (Fig. 1). All these species are morphologically diagnosable from each other and are restricted to various regions of Thailand with *L.belliana* being found in the south, central, and southeast, *L.rubritaeniata* in the northeast, and *L.ocellata* and *L.peguensis* in the extreme northwest (Fig. 1). These species appear to be ecologically and geographically isolated due to their different temperature and moisture thresholds due to the historical formation of rivers through Thailand over past 20,000 years (Promnun et al. 2021a).

The Khorat Plateau in northeastern Thailand (Fig. 1) is the remnant of the Cimmerian microcontinent and has been a center of endemism since the early Cretaceous (Fernandez et al. 2009; Ridd et al. 2011; Meynell 2017; Yang and Grote 2018; Shen and Siritongkham 2020). Extinct endemic species range from ray-finned fishes, sauropod dinosaurs, crocodiles, turtles, and fossil hominids which are sister to modern orangutans (Chaimanee et al. 2003, 2006 2015; Wongko et al. 2019; Manitkoon et al. 2022; Chaimanee and Jaeger 2023). Given the Khorat Plateau’s modern climate with extreme wet and dry seasons and its unique geology, this pattern of endemism is still seen in contemporary groups such as freshwater bivalves, melon flies, blunt-headed burrowing frogs, puddle frogs, land snails, mud snakes, keelback snakes, and wolf snakes (Tumpeesuwan et al. 2007; Stuart and Chuaynkern 2007; Tumpeesuwan and Tumpeesuwan 2014; Boontop et al. 2017; Muanta et al. 2019; Nonsrirach and Lauprasert 2019; Vogel and David 2019; Jirapatrasilp et al. 2021; Köhler et al. 2021; Bernstein et al. 2022). Unfortunately, despite the endemism of this area, there are currently few conservation measures in place to protect its unique historical and contemporary biodiversity. On two recent expeditions to the Khorat Plateau during the dry season we discovered a new sexually reproducing population of *Leiolepis* that is morphology distinct from all other sexual species. Additionally, this new population of *Leiolepis* appears to be ecologically restricted to the elevated rocky habitats of the Khorat Plateau and seems to have adapted to these rocky environments by having the ability to dig interconnected, subterranean burrows into and beneath large, isolated rock piles. This type of natural history is unknown for any population of *Leiolepis*, which as stated above, are all adapted to sandy or loose soils in coastal and inland grassland environments.

In this study we use a combination of morphological and molecular datasets to test the hypothesis that this new population of *Leiolepis* from the Khorat Plateau represents a distinct species from all other sexual species of *Leiolepis*. Additionally, we will use the results and examples from the literature to demonstrate the Khorat Plateau’s conservation importance and its unique biogeographic connections with other regions of Southeast Asia.

## ﻿Materials and methods

All *Leiolepis* specimens were collected, photographed, and had liver tissue samples preserved in 95% ethanol for subsequent DNA sequencing. All specimens were fixed in 10% formalin and subsequently transferred into 70% ethanol. Color notes were taken from living specimens and digital images of living specimens. Scale counts and measurements were taken with Mitutoyo digital calipers to the nearest 0.1 mm under a Nikon SMZ 1500 dissecting microscope on the right side of the body where appropriate. Characters were obtained from Darevsky and Kupriyanova (1993) and Grismer and Grismer (2010) with new characters found here for sexual species (Suppl. material 1). We examined 104 specimens from five of the six sexual species for 54 characters (Suppl. material 1).

Measurements taken were: Snout–vent length (**SVL**), taken from the tip of the snout to the vent; head length (**HL**), measured from the posterior end of the retroarticular process of the mandible to the tip of snout; head width (**HW**), measured at the widest part of the head anterior to the tympanum; head height (**HH**), measured from the ventral surface of the mandible below the center of the eye to the dorsal surface of the head posterior to the eye; rostrum height (**RH**), measured from the ventral surface of the mandible to the dorsal surface of the rostrum above the nares; height of ear (**HE**), measured from the ventral margin of the tympanic opening to its dorsal margin; distance between nares (**DN**), measured with the tips of the calipers inserted into the narial openings; forearm length (**FA**), measured from the wrist to the outer edge of the flexed elbow; brachium length (**BL**), measured from the elbow to the limb insertion on the body; axilla–groin length (**AG**), measured from the posterior margin of the forelimb insertion to the anterior margin of the hind limb insertion on the body; hind limb length (**FL**), measured from the knee to the limb insertion on the body; tibia length (**TIB**), measured from the ankle to the flexed knee; length of the first enlarged, subdigital lamella on the third toe (**ESL**), measured from its base to its apex; third toe length (**TE**) measured from the base of the third toe to the base of the claw; chest width (**CW**), taken across the chest between brachium insertions on the body; pelvis width (**PW**), taken across the pelvic region between the hind limb insertions.

Meristic characters counted were the number of supralabials (**SL**), counted from the first enlarged scale at the angle of the jaw to the first enlarge scale contacting the postrostral scale; the number of infralabials (**IL**), counted from the first enlarged scale at the angle of the jaw to the first enlarge scale contacting the postmental scale; the number of scales across the frontal bone between the midpoint of the supraorbital regions that are not a supraorbital scales (**FB**); the number of postrostral scales contacting the rostral scale (**PR**); the number of supraorbital scales across dorsal surface of supraorbital region (**SO**); the number of enlarged, keeled suboculars below the ventral margin of the orbit (**SBO**); the number of enlarged scales along the ventral angle of the mandible (**ESM**); the number of transverse gular scales between the fourth enlarged mandibular scales along the angle of the jaw (**GBESM**); the ventral margin of the rostral scale forming a straight line with ventral margin of the first supralabial or not (**VMR**); the number of enlarged, keeled scale rows across the forearm midway between the elbow and wrist (**KSFA**); the number of dorsal scales between the inner margins of the left dorsolateral stripe at the widest part of the dorsolateral stripe (**DSS**); the number of non-keeled scale across the ventral side of the tibia midway between the ankle (**STIB**); the number of enlarged, plate-like scales along the dorsal surface of the first toe (**L1T**); the number of enlarged, subdigital lamellae on third toe (**ESL3T**); the number of enlarged, subdigital lamellae on the fourth toe (**SL4T**); the number of enlarged, plate like scales along the dorsal surface of the fourth toe (**L4T**); the number of ventral scale across the belly contacting the apex of the umbilical scar (**VSB**); number of femoral pores per side (**FMP**); the number of transverse non-pore-bearing scales between pore-bearing-femoral scales (**NPBS**); the number of scales that make up width of the dorsal tail coloration posterior to the pelvis (**NST**).

Color pattern and categorical characters recorded were the presence (1) or absence (0) of a black banding or dark mottling in the pectoral region (**BY**); ventral pattern (**VM**), banded or mottling (1), spotted (2), or no coloration (3); presence (1) or absence (0) of pale colored spots on flanks (**SF**); presence (1) or absence (0) of a Y-shape marking on the nape of the neck (**YN**); the number of pale colored ocelli or spots between the dorsolateral stripes at widest part of the body (**NSS**); gular coloration (**GC**), white (1), blue and black (2), yellow and black (3), red (4), black and white (5); ventral color (**VC**), yellow (1), white (2), black and white (3), black and blue (4); the number of pale colored dorsal stripes (**DS**); flank color (**FC**), orange and black (1), orange (2), yellow and orange (3), black and white (4), grey (5); subcaudal coloration (**TC**), white (1) or red (2); presence (1) or absence (0) of a pale colored ventrolateral stripe contacting axillary region (**VNSA**); the number of black transverse bars on the flanks (**TB**); presence (1) or absence (0) of a pale colored ventrolateral caudal stripe (**CS**); presence (1) or absence (0) of thin, transverse, dorsal, caudal bars (**CTB**); presence (1) or absence (0) of a white eyespot at the center of the ocelli (**WCES**); color of plantar surfaces (**PC**), white (1), red and black (2), white and black (3); mottling coloration of the ventral surface of the legs (**BMFT**), black (1), red (2), white (0); and presence (1) or absence (0) of constricted tail base (**CTAB**).

### ﻿Species concept and phylogenetic analyses

In this study we adopt the general lineage concept (de Queiroz 2007) and hypothesize that monophyletic clades in a phylogeny are evolutionary independent due to a lack of genetic admixture between populations of closely related lineages (Barraclough et al. 2003; de Queiroz 2007). Therefore, we identify any newly collected populations from the Khorat Plateau that form a well-supported monophyletic group distinct from all others in the phylogeny as a lineage that could potentially be a distinct species.

For all phylogenetic analyses we used sequence data from 135 samples representing all 10 species including seven samples from the newly discovered population from the Khorat Plateau and four previously published studies (Grismer and Grismer 2010; Grismer et al 2014; Promnun et al. 2021a, 2021b). Genomic DNA was extracted from liver tissues with the Qiagen DNeasy tissue kit (Valencia, CA, USA). We used double-stranded PCR to amplify roughly 800 aligned bases of the mitochondrial ND2 gene region using the primers METF6 (AAGCAGTTGGGCCCATACC) and CO1R1 (AGRGTGCCAATGTCTTTGTGRTT) which are listed in Macey et al. (1997) and Grismer and Grismer (2010). Amplification of 25 μl PCR reactions were executed on an Eppendorf Mastercycler gradient thermocycler. Amplification of genomic DNA began with an initial denaturation for 2 min at 95 °C followed by 95 °C for 35 s, annealing at 50 °C for 35 s, and extension at 72 °C for 150 s with four seconds added to the extension per cycle for 32 cycles. PCR products were visualized using electrophoresis through a 1.2% agarose gel, marker 100 bp, 1X TAE and stained with Red- Safe Nucleic Acid Staining Solution and photographed under UV light of Geldoc system (Quantum CX5, Villber, France). Successful amplifications were purified using innuPREP Gel Extraction Kit (Analytik Jena, Germany). Cleaned PCR products were sent to Genewiz from Azenta Life Sciences for sequencing in both directions. The accuracy of sequences was ensured by incorporating negative controls and sequencing complementary strands. New sequences were combined with the mitochondrial datasets of Grismer and Grismer (2010), Grismer et al. (2014), Promnun et al. (2021a, 2021b), aligned into a single dataset using MUSCLE (Edgar 2004) in Geneious 6.1 (Kearse et al. 2012), and checked for nucleotide ambiguities. All new sequences were deposited in GenBank under accession numbers PP987908 to PP988006.

The resulting mtDNA dataset was used to estimate relationships among all 135 samples in RAxML 8.1.1 (Stamatakis 2014), unpartitioned, with the GTRGAMMA model for sequence evolution, and assessed branch support using the automatic rapid bootstrap function on the CIPRES Science Gateway (Stamatakis 2014; Miller et al. 2010). We complimented the maximum likelihood analysis with a Bayesian analysis in BEAST 2.63 (Bouckaert et al. 2019). We used bModelTest to select the model of evolution, a Relaxed Log Normal Clock for the clock model, a Yule Model for the tree model, and each analysis was run for 350 million generations logging every 15,000. The BEAST log files were visualized in Tracer v. 1.7.0 (Rambaut et al. 2018) to ensure effective sample sizes (ESS) were well above 200 for all parameters. Maximum clade credibility trees using mean heights at the nodes were generated using TreeAnnotator v. 1.8.0 (Rambaut and Drummond 2007) with a burn-in of 10%. Nodes with Bayesian posterior probabilities (BPP) of 0.95 and above were considered strongly supported (Huelsenbeck et al. 2001; Wilcox et al. 2002). We considered nodes with values with a posterior probability of 0.90–0.94 as well-supported.

### ﻿Statistical analyses

All statistical analyses were conducted using R Core team 2021. We employed a multiple factor analysis (MFA) using the R package FactorMineR (Husson et al. 2017) and visualized using the Factoextra package (Kassambara and Mundt 2017). The MFA was conducted on a concatenated data set comprised of 16 normalized morphometric (SVL, HL, HW, HH, RH, HE, DN, FA, BL, AG, FL, TIB, ESL, TE, CW, and PW), 18 meristic (SL, IL, FB, PR, SO, SBO, ESM, GBESM, KSFA, DSS, STIB, L1T, ESL3T, L4T, VSB, FMP, NPBS, and NST), and 17 categorical characters (TB, BY, VM, SF, YN, NSS, GC, VC, DS, FC, TC, CS, CTB, WCES, PC, CTAB, and BMFT). To remove potential effects of allometry in the morphometric characters, size was normalized using the following equation: X adj = log(X) – β[log(SVL) – log(SVL mean)], where X adj = adjusted value; X = measured value; β = unstandardized regression coefficient for each population; and SVL mean = overall average SVL of all populations in the R package GroupStruct (Thorpe 1975, 1983; Turan 1999; Lleonart et al. 2000; Chan and Grismer 2022). The morphometrics of each species are normalized separately and then concatenated so as not to conflate intra- with interspecific variation (Reist 1986). All data were scaled to their standard deviation to insure they were analyzed based on correlation and not covariance. A non-parametric permutation multivariate analysis of variance (PERMANOVA) in the package vegan 2.5-3 (Oksanen et al. 2020) was used to determine if the centroid locations and group clusters of each species/population from the MFA were statistically different from one another (Skalski et al. 2018) based on the load scores of dimensions 1–5. Using loading scores as opposed to raw data, allows for the incorporation of the categorical characters which cannot be run in a PERMANOVA untransformed. The analysis calculates a Euclidean (dis)similarity matrix using 20,000 permutations. Lastly, Analyses of variance (ANOVA) were conducted on meristic and normalized morphometric characters to search for the presence of statistically significant mean differences (p < 0.05) among species across the data set. Characters bearing statistical differences were subjected to a TukeyHSD test to ascertain which species pairs differed significantly from each other for those characters.

## ﻿Results

**Figure 2.**
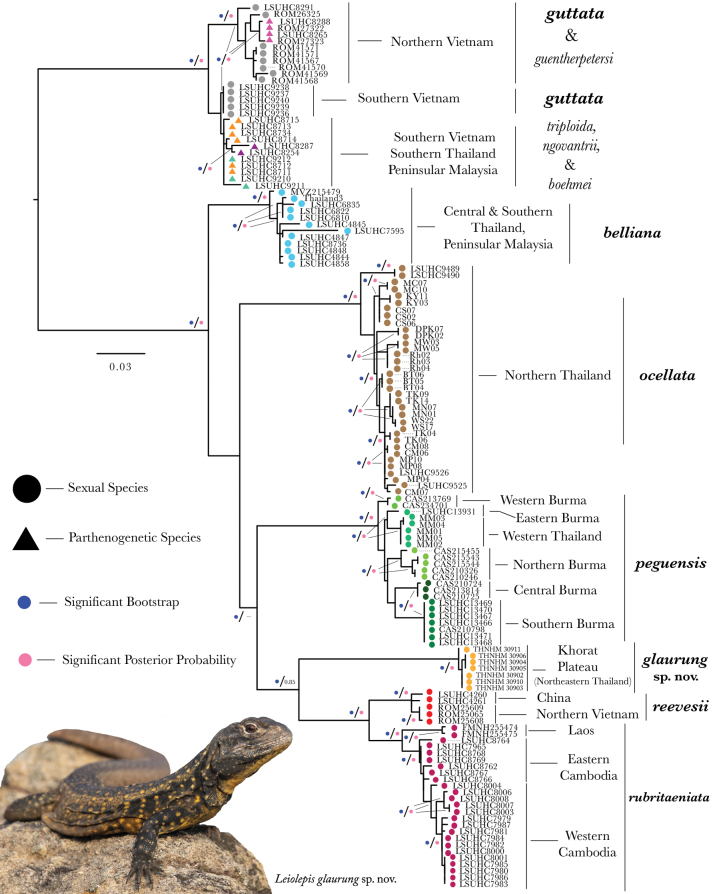
The mitochondrial gene tree for all 135 samples of *Leiolepis* from the Maximum Likelihood and Bayesian analyses.

All phylogenetic analyses returned matching topologies and near identical levels of nodal support (Fig. 2). The topology matches the relationships in the most recent nuDNA and mtDNA phylogenies of *Leiolepis* from Grismer et al. (2014) and Promnun et al. (2021b), respectively. All samples of rock-dwelling *Leiolepis* from the Khorat Plateau were recovered as a monophyletic group with strong support (bootstrap of 100) and lineage independence from all other species (Fig. 2). The Khorat Plateau clade is at the end of a long branch and sister to all populations of the sister species *L.rubritaeniata* and *L.reevesii* from eastern Indochina, with strong support (bootstrap of 100) in the RaxML and moderate support (posterior probability of 0.85) from the BEAST analyses (Fig. 2).

**Figure 3.**
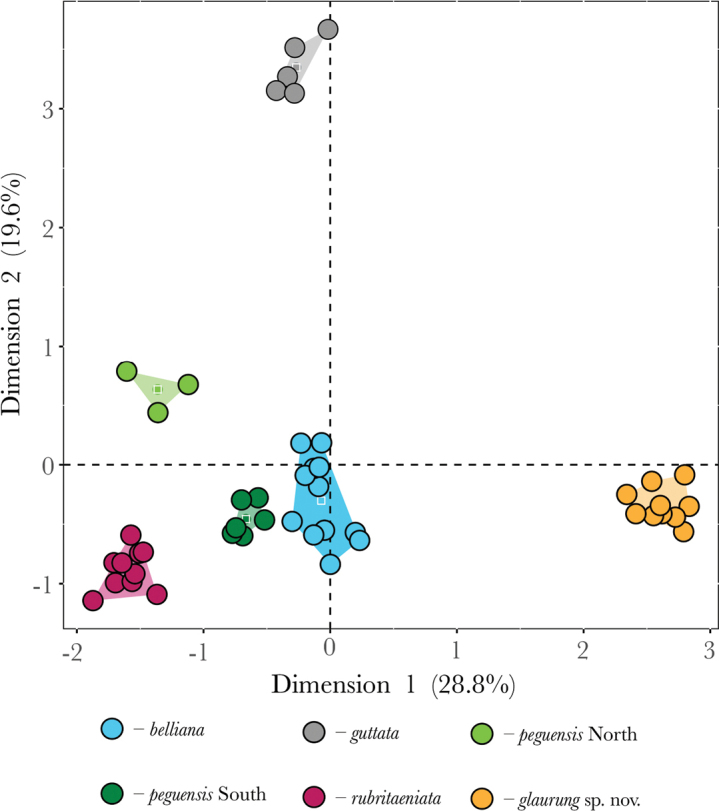
The clustering of well-supported clades along dimensions 1 and 2 from the MFA.

**Figure 4.**
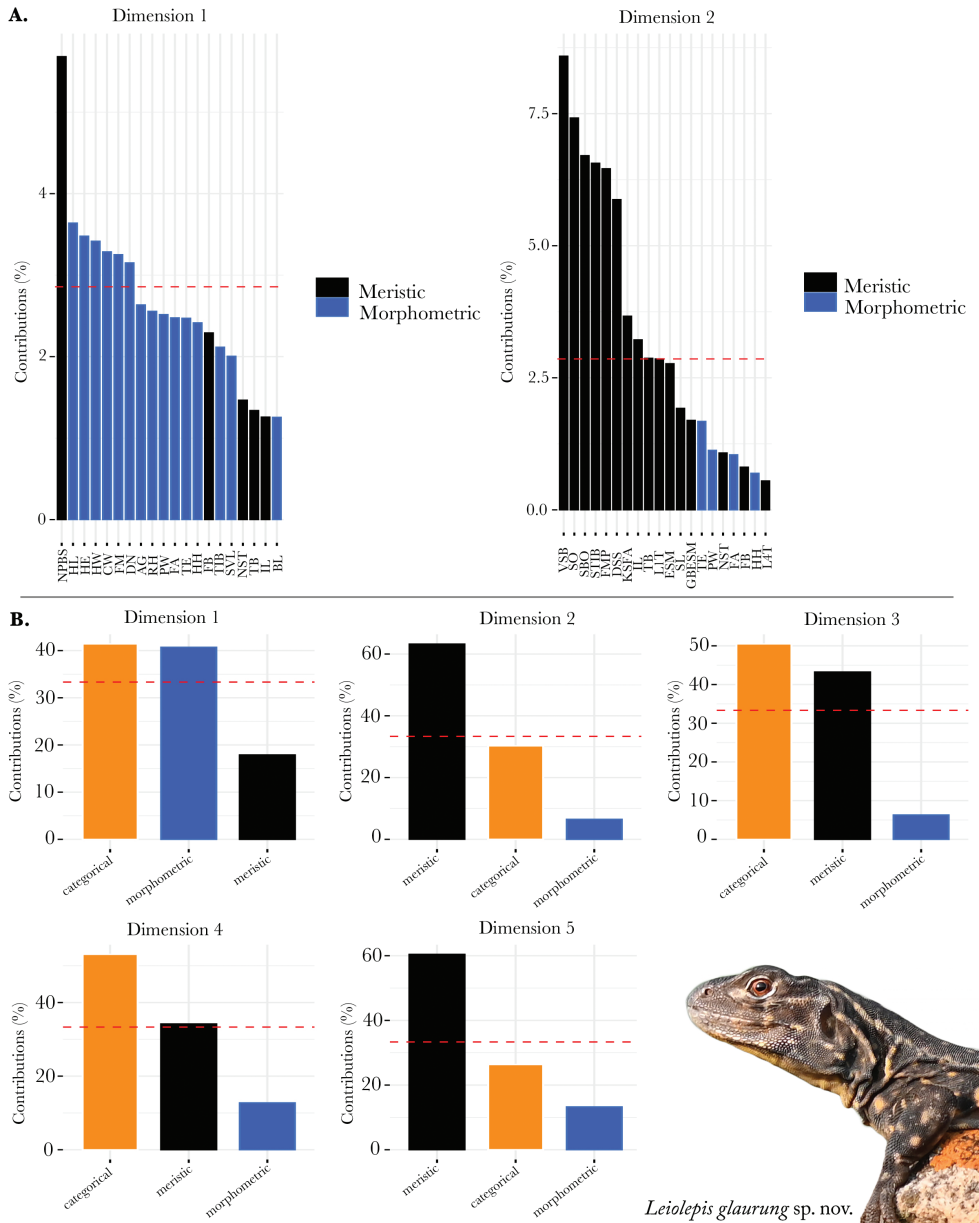
**A** The percent contribution of meristic and normalized morphometric characters of the first two dimensions of the MFA**B** The percent contribution of categorical, normalized morphometric, and meristic characters of the first five dimensions of the MFA.

The MFA plot shows that the Khorat Plateau samples cluster outside all other species, including the closely related species *L.rubritaeniata* and *L.reevesii* (Figs 2, 3). Dimension 1 accounted for 28.8% of the variation and dimension 2 accounted for 19.6% of the variation in the dataset (Fig. 3). Across the first five dimensions (D1–D5) the morphometric variables accounted for 40% (D1), 7% (D2), 7% (D3), 12% (D4), and 12% (D5) of the variability of those dimensions (Fig. 4). Likewise, for the first five dimensions the categorical data accounted for 40% (D1), 30% (D2), 50% (D3), 52% (D4), and 25% (D5) of the variation (Fig. 4). Lastly, the contributions of the meristic variables for the first five dimensions accounted for 17% (D1), 62% (D2), 43% (D3), 37% (D4), and 60% (D5) of the variation (Fig. 4).

The results from the PERMANOVA further demonstrate the morphological differences between Khorat Plateau *Leiolepis* and the other clades of sexual species (Table 1). Although our low sample size of *L.reevesii* (*n* = 4) restricted us from including it in our analyses, we expect *L.reevesii* to be statistically distinct from the Khorat Plateau samples as its morphology is similar to that of *L.rubritaeniata*, which was once a subspecies of *L.reevesii* (Böhme 2003). In addition, the ANOVAs and subsequent TukeyHDS tests demonstrated that the Khorat Plateau has statistically different combinations of mean values of AG, BL, CW, DN, FA, HE, HH, HL, HW, PW, TIB, TE, RH, DS, VSB, TB, NST, NPBS, KSFA, FMP, FB, STIB, L1T, and L4T from all other sexual species (Suppl. material 2). Given the morphological differences (Figs 3, 4; Table 1) and their independent phylogenetic position (Fig. 2), we hypothesize the Khorat Plateau population represents a new distinct sexual species of *Leiolepis*.

**Table 1.** Results from the PERMANOVA analysis comparing pairs of sexual species of *Leiolepis* bearing statistical mean morphospatial differences at the *p.*adjusted threshold based on the load scores of Dim1–Dim5 of the MFA. * = significant difference.

Clade comparisons	*p.*value	*p.*adjusted	sig
*L.glaurung* sp. nov. vs *L.rubritaeniata*	0.00004000	0.00083998	*
*L.glaurung* sp. nov. vs *L.belliana*	0.00002000	0.00041999	*
*L.glaurung* sp. nov. vs *L.peguensis* South	0.00022000	0.00461991	*
*L.glaurung* sp. nov. vs *L.peguensis* North	0.00409992	0.08609828	
*L.glaurung* sp. nov. vs *L.guttata*	0.00057999	0.01217976	*
*L.rubritaeniata* vs *L.belliana*	0.00002000	0.00041999	*
*L.rubritaeniata* vs *L.peguensis* South	0.00012000	0.00251995	*
*L.rubritaeniata* vs *L.peguensis* North	0.00309994	0.06509870	
*L.rubritaeniata* vs *L.guttata*	0.00035999	0.00755985	*
*L.belliana* vs *L.peguensis* South	0.00010000	0.00209996	*
*L.belliana* vs *L.peguensis* North	0.00179996	0.03779924	*
*L.belliana* vs *L.guttata*	0.00025999	0.00545989	*
*L.peguensis* South vs *L.peguensis* North	0.01179976	0.24779504	
*L.peguensis* South vs *L.guttata*	0.00209996	0.04409912	*
*L.peguensis* North vs *L.guttata*	0.01785714	0.37500000	

### ﻿Taxonomy

#### ﻿ Leiolepis glaurung sp. nov.

Taxon classificationAnimaliaSquamataAgamidae

0E624381-AF83-5F10-996C-1D54D8DA7038

https://zoobank.org/22E2BD02-9672-434B-B4DF-43AD636677B7

**Figure 5.**
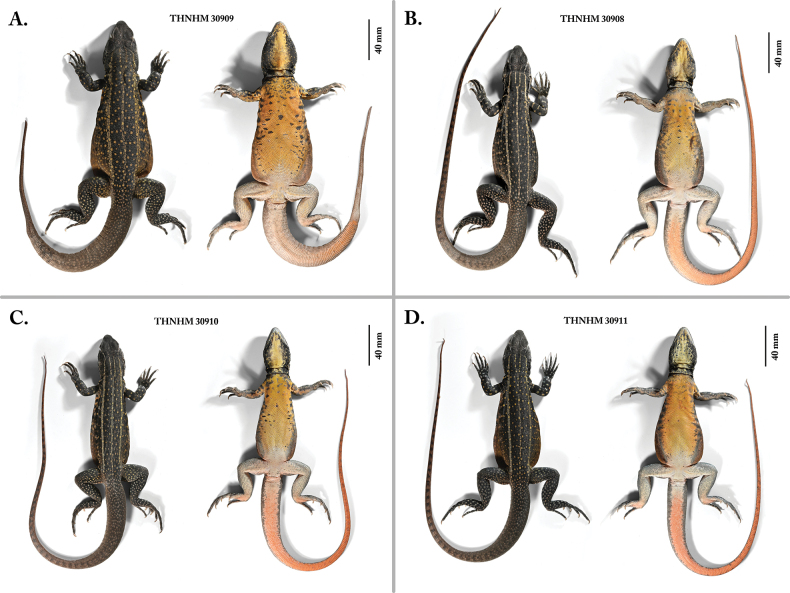
Dorsal and ventral views of the holotype and paratypes of *Leiolepisglaurung* sp. nov. **A** male holotype THNHM 30909 **B** female paratype THNHM 30908 **C** male paratype THNHM 30910, and **D** male paratype THNHM 30911.

**Figure 6.**
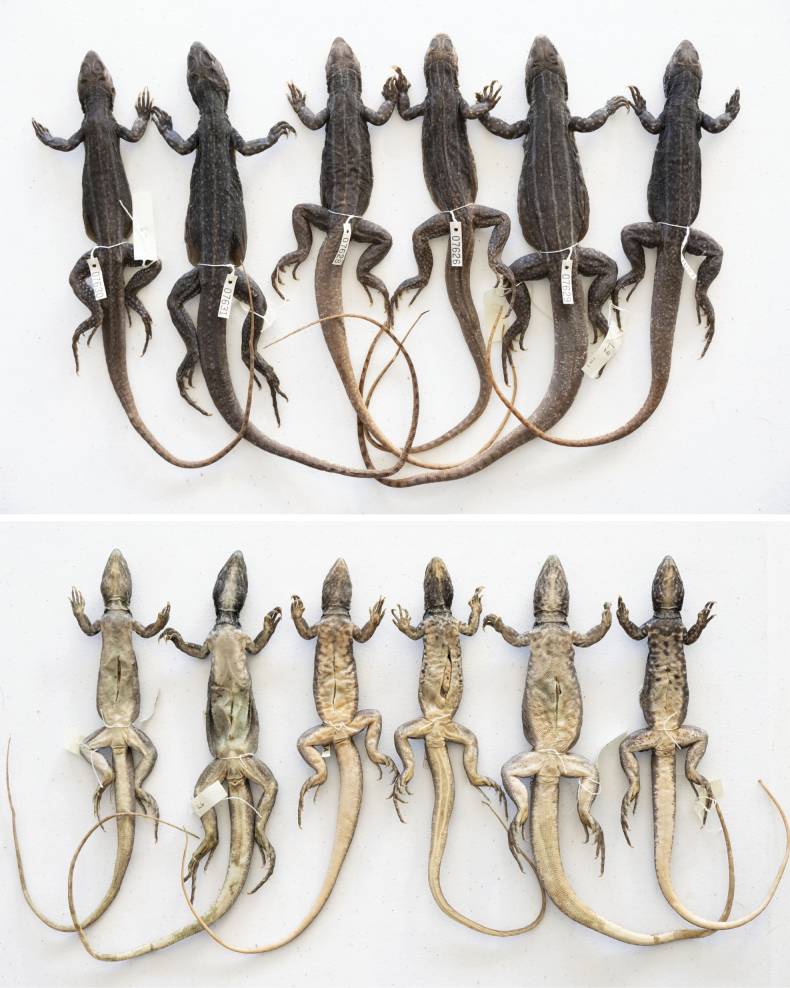
Dorsal and ventral views of paratypes THNHM 30906, THNHM 30907, THNHM 30904, THNHM 30902, THNHM 30905, and THNHM 30903 *Leiolepisglaurung* sp. nov. Specimen tags bear field numbers.

Figs 5 , 6 , 9 , 11 ; Table 1 ; Suppl. materials 1 , 2 Suggested English name: Khorat Plateau Butterfly Lizard

##### Type material.

***Holotype*.** Adult male (THNHM 30909; Fig. 5A) collected from just northeast of Wat Phu Noi in Kaeng Kheng Subdistrict, Kut Khaopun District, Ubon Ratchathani Province, Thailand (15°48'10.7"N, 105°09'24.9"E) on 20 March 2023, at 10:00 am by Pratyaporn Wanchai, Anchalee Aowphol, Attapol Rujirawan, Akrachai Aksornneam, Jesse L. Grismer, L. Lee Grismer, Evan S. H. Quah, and Matthew L. Murdoch. ***Paratypes*.** Adult female (THNHM 30908; Fig. 5B) and two adult males (THNHM 30910–30911; Fig. 5C, D) bear the same locality and collectors as the holotype. Three adult males (THNHM 30902–30903, THNHM 30907) and three adult females (THNHM 30904–30906; Fig. 6) were collected by Pratyaporn Wanchai between 4–10 July 2020 from the same locality as the holotype.

##### Diagnosis.

**Figure 7.**
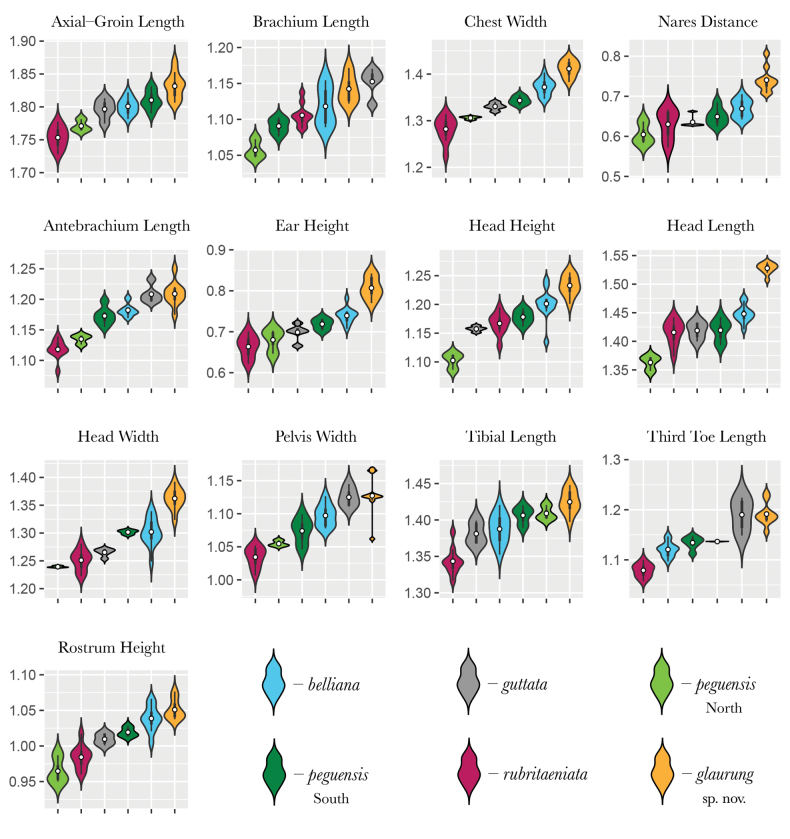
Comparisons of the normalized morphometric characters showing the ranges, frequencies, mean (white dots), and 50% quartiles represented by black bars.

**Figure 8.**
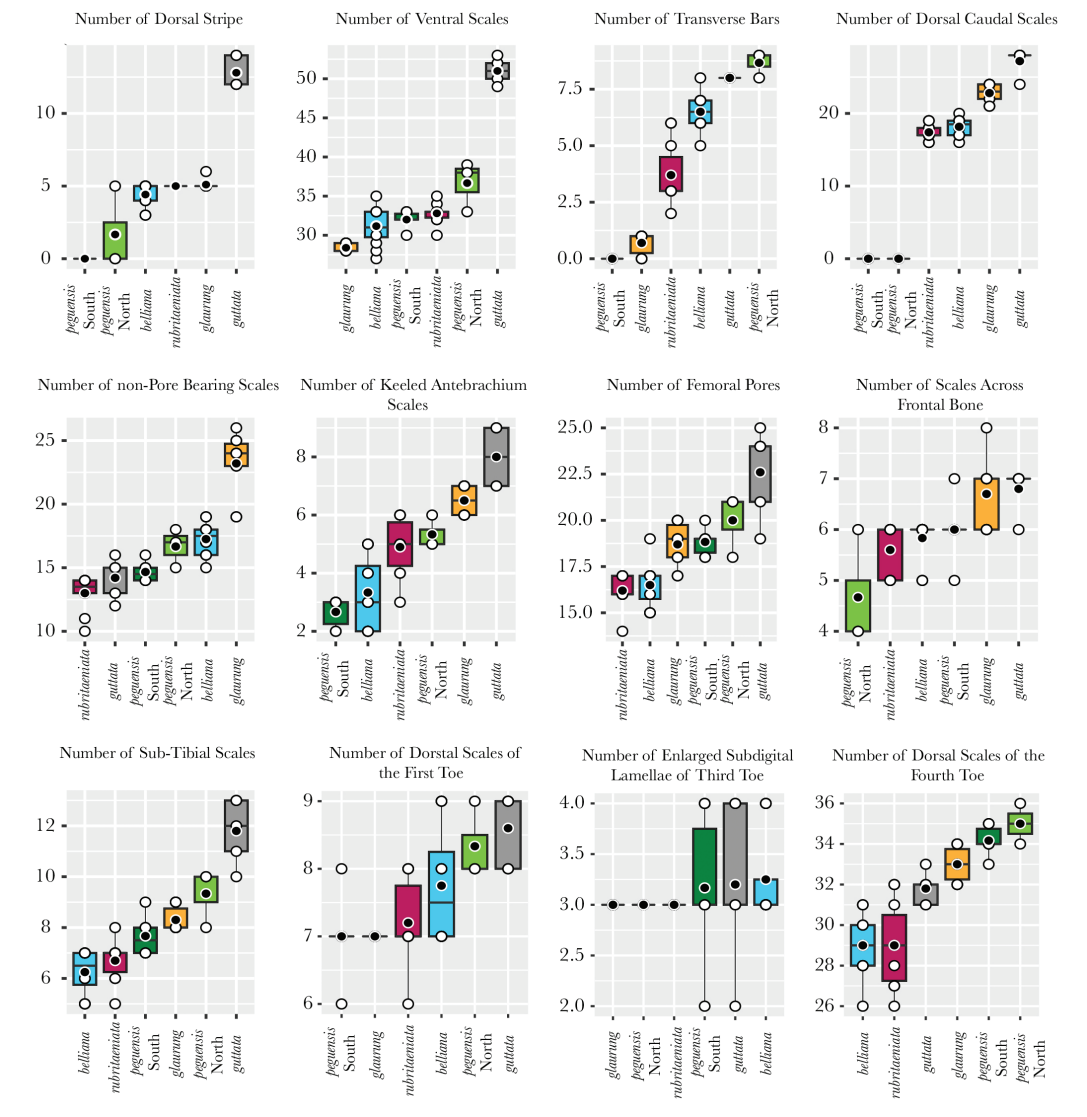
Comparisons of statistically different meristic characters among well-supported clades of *Leiolepis*. White circles are means and the black horizontal bars are medians.

*Leiolepisglaurung* sp. nov. can be diagnosed from all sexual species of *Leiolepis* by having a black gular region with a wide medial yellow stripe, a yellow ventrum with black mottling, bright red to orange ventral tail coloration, having reduced to no expandable flanks, and having a maximum of one black transverse bar on the flank (Suppl. material 1). *Leiolepisglaurung* sp. nov. can be further diagnosed from all other sexual species by having the combination of a AG of 61.0–88.0 mm; BL of 11.0–16.8 mm; CW of 22.3–33.1 mm; DN of 4.3–6.7 mm; FA of 14.4–19.0 mm; HE of 5.6–7.1 mm; HH of 14.0–21.3 mm; HL of 28.8–41.9 mm; HW of 19.2–29.7 mm; PW of 10.5–17.0 mm; TIB of 24.34–29.0 mm; TE of 14.0–18.0 mm, RH of 9.7–14.3 mm; three dorsal stripes; 28–29 ventral scales; 21–24 dorsal caudal scales; 19–26 non-pore bearing scales between the pore-bearing femoral scales across the pelvis; maximum number of seven keeled scale rows across the forearm; 17–20 femoral pores per side; 6–8 scales across the frontal bone; maximum of nine subtibial scales; seven scales long the dorsal surface of the first toe; three enlarged subdigital lamellae on the third toe; and 32–34 scales along the dorsal surface of the fourth toe (Figs 7, 8).

##### Description of holotype.

Head large, (HL 41.9 mm; HL/SVL 0.25) obtusely rounded in lateral profile, triangular in dorsal profile (HW 29.7 mm; HW/HL 0.71); interorbital, frontal region, and rostrum, convex (HH 21.3 mm; RH 14.3 mm; RH/HH 0.67), sloped anteriorly, covered with small, undifferentiated keeled scales; occipital and supraorbital regions covered with 19 keeled, granular scale rows half the size as dorsal head scales; canthus rostralis short, rounded; dorsal head scales strongly keeled, eight keeled scales across the frontal bone between supraorbital regions; rostral large, triangular (wider than long), bordered posteriorly by six smaller scales; external nares large, set wide apart (DN 6.7 mm; DN/HW 0.23) rounded, directed laterally, set in single, oval, nasal scale surrounded by several small scales; elongate, keeled, large fused, suborbitals (on five right side; five on the left) extend from anterior margin of eye to posterior margin of eye; superciliary scales elongate, keeled, imbricate, continuous with canthal scales; eyelid scales granular; tympanum naked, deeply set, surrounded by granular scales; temporal scales keeled, small, slightly raised; nine rectangular supralabials whose contact with one another produces an distinct labial margin, bordered ventrally by small granular scales; mental longer than wide, pointed posteriorly, larger than adjacent infralabials; two large postmentals in contact medially, being first of a series of 14 enlarged scales along the angle of jaw (left side: right side was damaged); 10 rectangular infralabials; gular scales small, rounded, granular; two distinct anterior and posterior gular folds present; dewlap absent; antehumeral fold continuous with posterior gular fold (Figs 5A, 6A).

Body elongate (AG 88.0 mm; AG/SVL 0.52) somewhat dorsoventrally compressed; expandable flanks reduced to absent; body scales small, granular, slightly keeled; 43 scales between dorsolateral stripes; 20 scales between vertebral stripe and dorsolateral stripes at widest point of trunk; scales of flanks abruptly transition into much larger, flat scales of belly and pectoral region; 28 scales across middle of belly contacting apex of the umbilical scar; precloacals smooth and much smaller than ventral scales; forelimbs short, robust (FA 19.0 mm; BL 16.8 mm); dorsal surface of forelimbs and posterior surface of brachia covered with large, keeled, imbricate scales; six rows of enlarged, keeled scales across forearm; ventral surface of forelimbs covered with granular scales; plantar scales small, granular; subdigital lamellae of fingers are composed of a single wide transversely elongated scales; claws long; hind limbs relatively long (FM 33.6 mm; TIB 27.2 mm); dorsal scales on hind limbs small, weakly keeled; scales on anterior surface of thighs large, flat, weakly keeled, imbricate; those on forelegs slightly enlarged, keeled; postfemoral scales small, granular; eight longitudinal rows of large, smooth, flat, imbricate subtibial scales; 36 total femoral pores; each pore set in larger scale; 25 non-pore-bearing scales between pore-bearing-femoral scales across the pelvis; plantar scales small, raised; subdigital lamellae of toes bicarinate, 32 enlarged, plate-like scales along dorsal surface of the fourth toe; three enlarged, triangular scales on posterior surface at base of third toe (ESL 2.6 mm; ESL/TE 0.16); seven enlarged, plate-like scales along length of first toe; tail dorsoventrally compressed, noticeably wider at base, constricted at its contact point with body, covered dorsally with small, keeled scales grading ventrally into larger, flat, weakly keeled, subcaudals; caudal scales in transverse rows encircle the tail; and the last 110 mm of the tail is regenerated (Fig. 5A).

##### Coloration in life.

**Figure 9.**
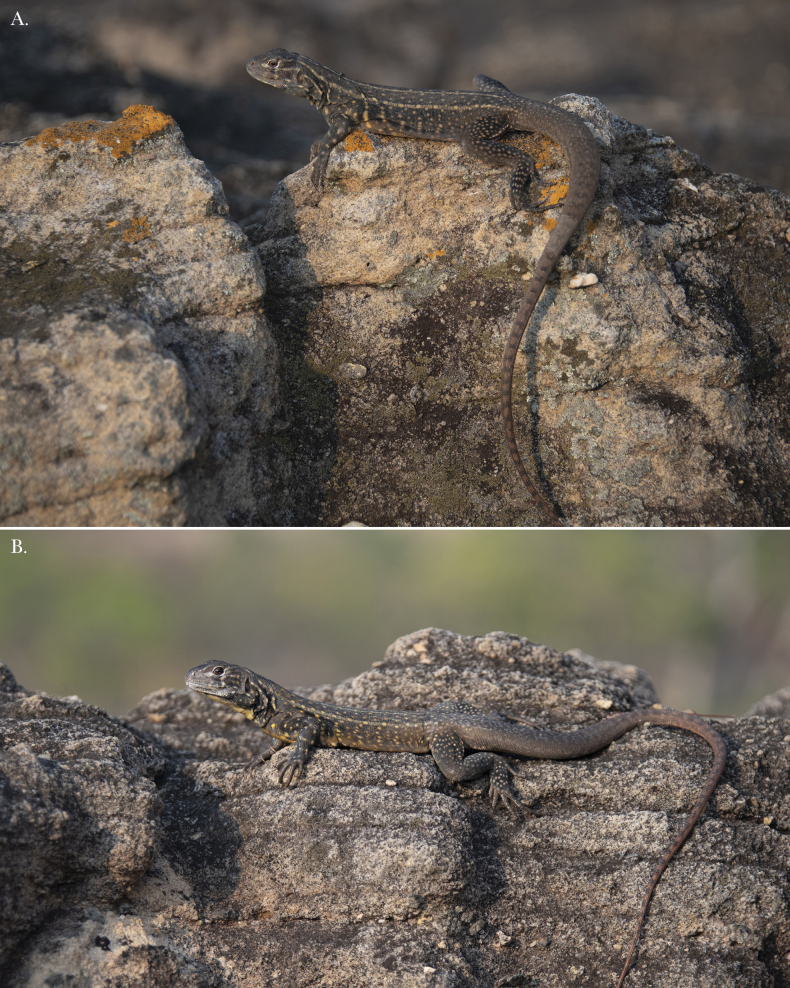
**A** Female paratype THNHM 30908 **B** male paratype THNHM 30911 of *Leiolepisglaurung* sp. nov.

Dorsal ground color of head, body, limbs, and tail is grey to almost black (when animal is cold base color is black); anterior portion of the head pale grey with no pattern; three white lines radiate from the posterior region of the orbit with one extending posteriorly to the parietal region, one to the tympanic region, one to the corner of the mouth; four yellow stripes extending posteriorly from the parietal region of the head, the two central stripes connect on the nape of the neck forming a yellow Y-shape and extend posteriorly as a vertebral stripe that terminating at the anterior margin of the pelvis, the two lateral stripes extend dorsolaterally from the parietal region running the entire length of the body terminating at the anterior margin of pelvis; dorsal pattern is composed of three yellow dorsal stripes (mentioned above) separated by distinct darker regions with yellow spots; the base color of the flanks are yellowish orange with one black transverse bar in the axillary region, followed by pale yellow transverse bands composed of small yellow spots, three (L) and four (R); the forearms have prominent yellow and white spots; the hind limbs have small white ocelli with diffuse edges; the dorsal caudal coloration is composed of the same small ocelli as on the hind limbs and the coloration turns into dark transverse caudal bars approximately 30% down the length of the tail; the gular region is black with a wide medial yellow strip; pectoral region is yellow with black mottling; the limb ventral color is yellow; subcaudal coloration is bright red to orange and extends laterally to the dorsolateral coloration of the tail (Figs 5A, 6, 9, 11)

##### Etymology.

The specific epithet *glaurung* is in reference to the large, terrestrial, golden-colored, non-winged dragon, Glaurung in Middle-earth – a character created by J.R.R. Tolkien in *The Silmarillion* (1977). Glaurung the Golden is the father of all dragons and tunneled into the sides of mountains forming burrows. The reduced expandable lateral flanks, yellow ventral and dorsal colors, with the construction of burrows beneath rocky outcrops is similar to the descriptions of Glaurung mentioned above, from ‘The Silmarillion’ and ‘The Children of Húrin’ (Tolkien 1977, Tolkien and Tolkien 2007). Additionally, in Thailand the word “Yae” is used to refer to *Leiolepisbelliana*, *L.ocellata*, and *L.rubritaeniata*. However, on the Khorat Plateau the populations of *Leiolepisglaurung* sp. nov. are called “Yarb”.

##### Distribution.

*Leiolepisglaurung* sp. nov. is currently known from three locations on the Khorat Plateau (Fig. 1). We have only collected specimens from the type locality in Ubon Ratchathani Province (Fig. 1) from the Phu Phan Formation (Jin-geng and Meesook 2013), however a population from Chaiyaphum Province (15°54'7.92"N, 102°9'50.4"E) in the western region of the Khorat Plateau was observed and photographed, but no specimens were collected (AK, AR, and PP pers. obs.), and from southern extent of the plateau on the border of Cambodia (PW pers. obs.) in Ubon Ratchathani Province (Fig. 1). Lizards from both populations are very similar in appearance to those from the type locality.

##### Variation.

Differences in scale counts and measurements are presented in Suppl. material 1. The coloration of the males THNHM 30911 and THNHM 30910 are similar to the holotype but their subcaudal coloration is a much more vibrant coral-red and they have complete original tails (Fig. 5C, D).The coloration of the yellow stripes and spots that composed the dorsal pattern, and the flank coloration are not as pronounced in the female specimens THNHM 30904–30906 and 30908 as they are in the males (Figs 6, 9; Suppl. material 1). Additionally, the dorsal caudal pattern is only faintly visible in the female specimens (Figs 6, 9).

##### Natural history.

**Figure 10.**
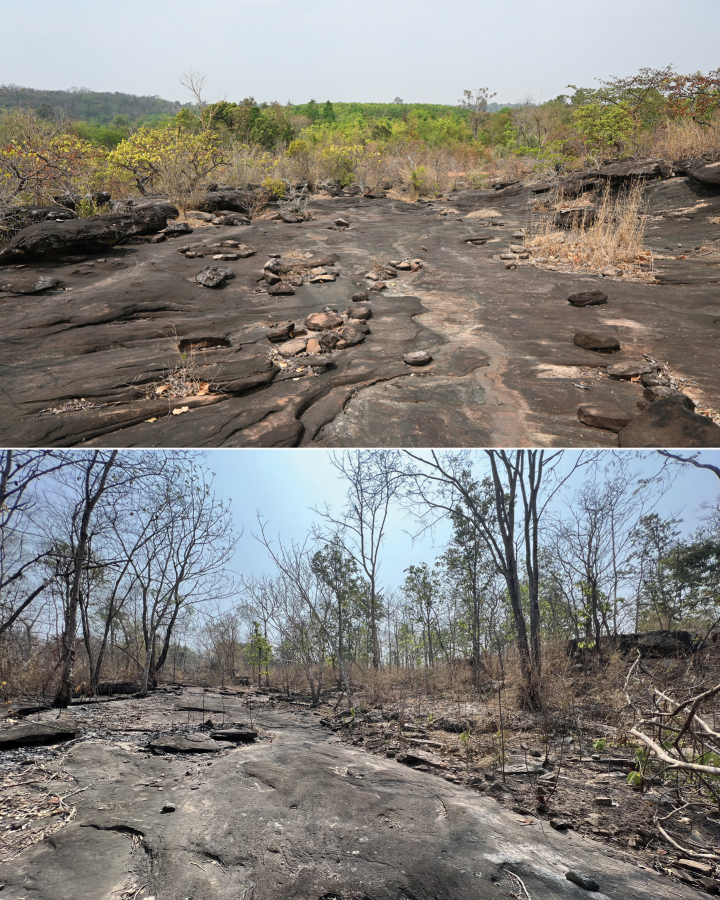
The rocky habitat of the Khorat Plateau from the type locality.

**Figure 11.**
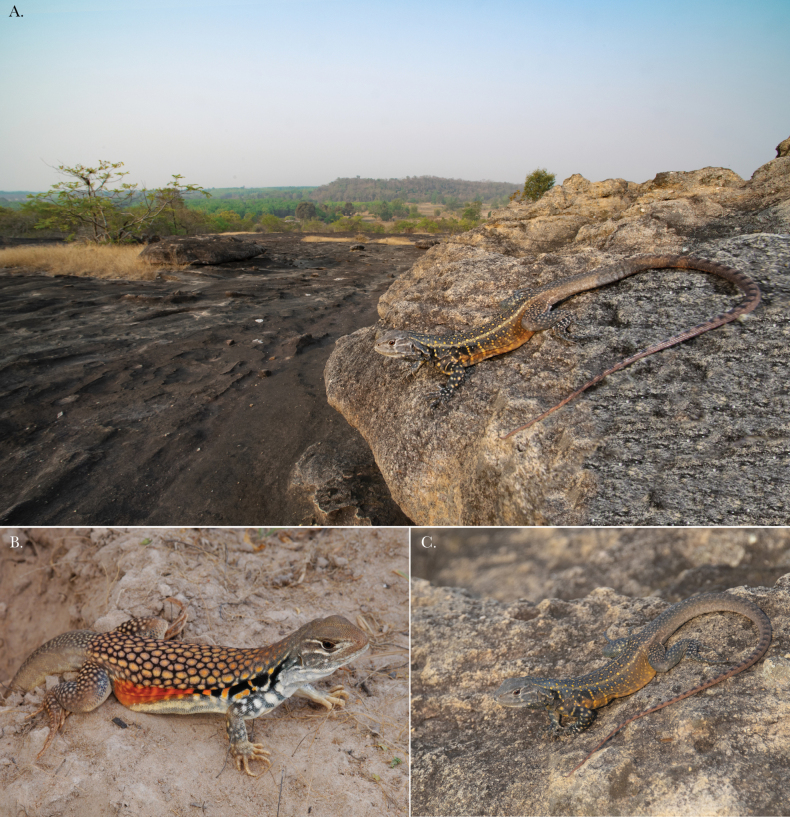
**A** Male paratype THNHM 30911 displaying the dorsal and lateral display colors and its rocky habitat in the background **B** adult male *Leiolepisrubritaeniata* from southeastern Cambodia **C** male paratype THNHM 30911 of *Leiolepisglaurung* sp. nov.

**Figure 12.**
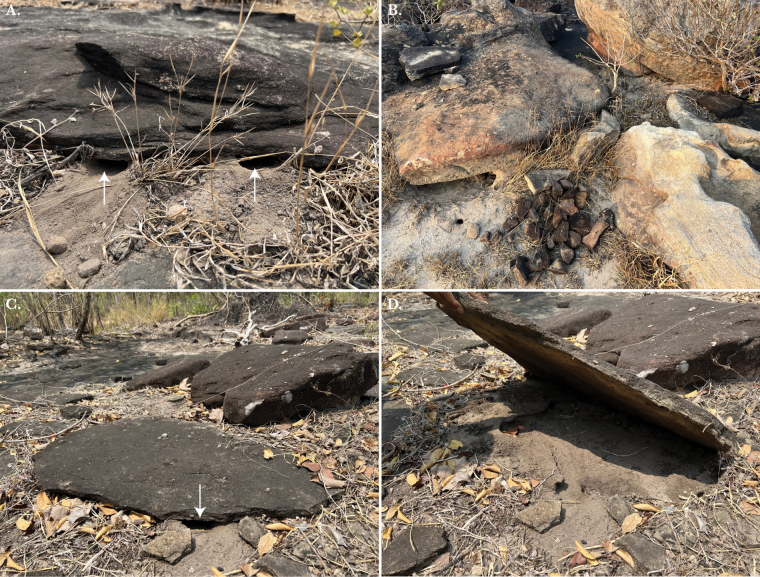
**A** Two burrow entrances going beneath a large rock **B** multiple small burrow entrances in the soil underneath and around a rock pile **C** a single entrance hole to a burrow beneath a large rock **D** the rock from panel C lifted to show the shallow subterranean burrow of an individual *Leiolepisglaurung* sp. nov.

Similar to other species of *Leiolepis*, *Leiolepisglaurung* sp. nov. constructs subterranean tunnels. However, given that they live exclusively in rocky habitats, individuals make compressed and shallow burrows in patches of loose soil underneath rocks or rockpiles (Figs 10–12). Males tend to forage during the hottest part of the day and the feces we found appeared to be mostly composed of vegetation and arthropods. *Leiolepisglaurung* sp. nov. is a food source for local people in the area (as many *Leiolepis* species are across Indochina; Grismer and Grismer 2010) and talking with local collectors, they report that individuals are highly philopatric. The collectors say that if the burrow is open that means they are out foraging and if the burrow is plugged with soil they are inside. Additionally, local people say that *Leiolepisglaurung* sp. nov. is mostly active during the dry season (November–March) and estivates during the rainy season (April–November) but will come out of their burrows after rains to eat arthropods. Lastly, all individuals observed and collected (specimens THNHM 30908–30911) were found during the day at 36 °C and 77% humidity. Other species of reptiles observed sharing the same habitat at the type locality included *Calotesversicolor* (Daudin, 1802), *Dixoniussiamensis* (Boulenger, 1899), *Gekkopetricolus* Taylor, 1962, *G.gecko* (Linnaeus, 1758), *Scincellamelanosticta* (Boulenger, 1887), *S.rupicola* (Smith, 1916), *Eutropismacularia* (Blyth, 1853), *Chrysopeleaornata* (Shaw, 1802), *Lycodonlaoensis* Günther, 1864 and *Calloselasmarhodostoma* (Kuhl, 1824).

## ﻿Discussion

Our results indicate that the samples of *Leiolepis* from the Khorat Plateau reported here are phylogenetically, ecologically, and morphologically distinct from all other sexual species of *Leiolepis* and as such, represent a new species. We hypothesize that *Leiolepisglaurung* sp. nov. has undergone multiple morphological adaptions to balance living in a rocky environment with pressures of sexual selection. Others have demonstrated that the color combination and expandable flanks of male *Leiolepis* are important for courtship and antagonistic confrontations and are likely to be under some degree of sexual selection (Hartmann et al. 2012; Grismer et al. 2014). Grismer et al. (2014) further hypothesized that the amount and combination of colors on the expandable flanks are what drove the hybridization events that created the parthenogenetic species of *Leiolepis*. *Leiolepisglaurung* sp. nov. is distinct from all other sexual species in that individuals retain the bright display colors on the flanks (Fig. 11) but appear to have reduced or lost the ability to expand the flanks. We hypothesize this is an adaptation to reduce their body diameter to allow access into the smaller burrows beneath rocks in shallow condensed soils (Fig. 12), unlike the larger and deeper burrows constructed in looser soils in savannahs and coastal areas by other *Leiolepis* species in Indochina. As a result of the reduced expandable lateral display area, we hypothesize that *Leiolepisglaurung* sp. nov. has evolved contrasting brightly colored subcaudal, ventral, and gular regions (Fig. 5) to act as secondary sexual displays. Given the dark base color of the dorsum, these colors and associated displays would possibly stand out to females against the dark color of the substrate (Fig. 10). Field observations indicate that *Leiolepisglaurung* sp. nov. lives adjacent to *L.rubritaeniata* which occupies the intervening sandy savannah habitats in the area and appear to not interact with each other. We hypothesize that the unique ecology and display coloration between the two species (Fig. 11) has kept these two species genetically isolated (Fig. 2). Additionally, these ecological and morphological differences discussed above between them has led to local collectors calling them different names – “Yarb” for *Leiolepisglaurung* sp. nov. and “Yae” for *L.rubritaeniata*, *L.belliana*, and *L.ocellata*.

Our field work indicates that *Leiolepisglaurung* sp. nov. is endemic to the Khorat Plateau (Fig. 1) further highlighting the growing pattern of endemism in this unique region of Thailand. The Khorat Plateau is broadly composed of moist deciduous forest and the environment drastically changes from the dry season to the rainy season. During the rainy season, regions of the plateau flood as the Mun and Chi rivers swell and drain the plateau into the southern Mekong River, creating additional unique microhabitats only available during the wet season. Most of the contemporary endemic species or genetic lineages on the Khorat Plateau are from groups such as freshwater bivalves, land snails, frogs, and water snakes that are most active during the wet season (Tumpeesuwan et al. 2007; Muanta et al. 2019; Jirapatrasilp et al. 2021; Köhler et al. 2021; Bernstein et al. 2022). However, *Leiolepisglaurung* sp. nov. is most active during the dry season and may be a good bioindicator that there are other undescribed species active only during this time of the year. This could mean that there are groups of species adapted to either the wet or dry season on the Khorat Plateau.

Lastly, irrespective of annual activity times, the endemic species and lineages on the Khorat Plateau are generally sister to species or populations from either Sundaland or Indochina (Fernandez et al. 2009; Boontop et al. 2017; Muanta et al. 2019; Jirapatrasilp et al. 2021; Bernstein et al. 2022; Chaimanee and Jaeger 2023). *Leiolepisglaurung* sp. nov. mirrors this pattern being recovered as sister to the remaining populations of *Leiolepis* east of the Chao Phraya River (Figs 1, 2). These conflicting biogeographic patterns indicate that during its formation, the Khorat Plateau may have been colonized multiped times from different regions of Sundaland and Indochina. These biogeographic connections may be the key to understanding why the species composition on the Khorat Plateau has historically been and currently is a mixture of common, wide-ranging, and locally endemic species.

The Khorat Plateau needs further biodiversity surveys during the wet and dry seasons to (1) obtain an understanding of which species are active at what times of the year; and (2) to collect additional specimens and genetic samples for taxonomic and broader biogeographic studies. These types of data would be vital to forming effective conservation measures to provide some level of protection for the unique species and ecosystems of the Khorat Plateau.

## Supplementary Material

XML Treatment for Leiolepis glaurung

## ﻿Additional information

### Author ORCIDs

Attapol Rujirawan https://orcid.org/0000-0001-9179-6910

Matthew L. Murdoch https://orcid.org/0000-0001-5914-6408

Akrachai Aksornneam https://orcid.org/0000-0003-4780-376X

Evan S. H. Quah https://orcid.org/0000-0002-5357-1953

L. Lee Grismer https://orcid.org/0000-0001-8422-3698

Jesse L. Grismer https://orcid.org/0000-0002-2542-5430

Anchalee Aowphol https://orcid.org/0000-0001-9504-4601

### Data availability

All of the data that support the findings of this study are available in the main text or Supplementary Information.

## Supplementary materials

Supplementary material 1 Raw meristic, categorical, and color pattern data from all specimens of *Leiolepis* used in this study Data type xlsx Explanation note m = male; f = female; - = data not taken; NA = specimen was damaged, and character could not be taken. https://binary.pensoft.net/file/1122043This dataset is made available under the Open Database License (http://opendatacommons.org/licenses/odbl/1.0/). The Open Database License (ODbL) is a license agreement intended to allow users to freely share, modify, and use this Dataset while maintaining this same freedom for others, provided that the original source and author(s) are credited.Pratyaporn Wanchai, Attapol Rujirawan, Matthew L. Murdoch, Akrachai Aksornneam, Pattarapon Promnun, Amanda Kaatz, Jeren J. Gregory, Eddie Nguyen, William Van Iderstein, Evan S. H. Quah, L. Lee Grismer, Jesse L. Grismer, Anchalee Aowphol

Supplementary material 2 Significant adjusted *p*-values from the results of the ANOVA and TukeyHDS analyses comparing all pairs of sexual species of *Leiolepis* for all morphological characters Data type xlsx https://binary.pensoft.net/file/1122044This dataset is made available under the Open Database License (http://opendatacommons.org/licenses/odbl/1.0/). The Open Database License (ODbL) is a license agreement intended to allow users to freely share, modify, and use this Dataset while maintaining this same freedom for others, provided that the original source and author(s) are credited.Pratyaporn Wanchai, Attapol Rujirawan, Matthew L. Murdoch, Akrachai Aksornneam, Pattarapon Promnun, Amanda Kaatz, Jeren J. Gregory, Eddie Nguyen, William Van Iderstein, Evan S. H. Quah, L. Lee Grismer, Jesse L. Grismer, Anchalee Aowphol
